# The Role of Ureteral Stents for All Ureteroneocystostomies in Kidney Transplants

**Published:** 2011-05-01

**Authors:** M. R. Laftavi, Q. Chaudhry, R. Kohli, L. Feng, M. Said, K. Paolini, M. Dayton, O. Pankewycz

**Affiliations:** 1 *Department of Surgery, Division of Transplantation,*; 2 *Department of Medicine, State University of New York (SUNY) at Buffalo, Buffalo, NY, USA*; 3 *Division of Transplantation, Kaleida Health, Buffalo, NY, USA*

**Keywords:** Ureter, Ureteral stents, Renal transplantation, Ureteral anastomosis

## Abstract

Background: Despite significant advancements in renal transplantation, certain basic surgical practices such as the routine use of ureteral stents (US) remain controversial. A recent met-analysis of ureteral stenting concluded that the routine use of US resulted in improved outcomes. In contrast, the indiscriminate use of US can lead to adverse complications.

Objective: To better define this question, we reviewed our single center experience in which US were placed selectively.

Methods: 301 patients were eligible to be enrolled. 55 living donor and 246 deceased-donor charts were analyzed for donor and recipient clinical characteristics, immunosuppressive therapy and outcomes.

Results: 28 US were placed for either small bladder capacity (n=7), unhealthy appearing bladder tissue (n=8) or for an uncertain vascular supply to the ureter (n=13). Patients with US did not develop urinary leaks, 8 (28%) developed complications including obstruction, encrustation, and urinary tract infections. 12 (4.3%) non-stented patients developed a clinically significant urinary leak. Risk factors for urinary leaks included dual and en-bloc pediatric donor kidney transplants, extended criteria donors and the use of single U stitch technique for ureteral anastomoses.

Conclusion: Our results demonstrate that the majority of patients can be successfully transplanted without the routine use of US. Selective use of US should be reserved for high-risk situations.

## INTRODUCTION

Due to the continued improvements in renal transplantation surgery and the evolution of modern immunosuppression therapeutic strategies, surgical complications of renal transplantation have been reduced dramatically. Urological complications of renal transplantation fell from 30% in the early reports of renal transplantation [1-3] to 2%–10% noted in more recent publications [4-6]. Many factors probably played significant roles in this improvement including the use of extravesicular techniques (Lich-Gregoire) for most cases and the significant reduction in steroid doses. However, certain issues such as the routine use of ureteral stents (US) remain controversial. 

There are many potential benefits of ureteral stenting including decompression of the ureter to avoid anastomotic tension, better ureteral alignment to avoid ureteral kinking or twisting and protection from ureteral narrowing or post-operative lumen obstruction due to mucosal edema or external compression. However, opponents of ureteral stenting argue that the stent itself can cause urinary obstruction by occluding the ureteric lumen or by dislodgement and migration. Moreover, US can exacerbate long-term strictures at the anastomotic site and may cause an erosion of the ureteral lumen leading to hematuria. Other potential complications of US include an increase in post-operative urinary infections [7-9], stent calcification [10-12], worsening post-operative pain and urinary urgency that can negatively impact on life quality [13-18]. The use of US also adds to overall costs of transplantation and requires another invasive procedure for removal.

A recent meta-analysis of 160 articles evaluating the use of US after renal transplantation concluded that the routine use of US results in fewer urinary complications [19]. However, the majority of papers comprising this meta-analysis did not report on or control for important factors that may predispose to the development of post-transplant urinary complications such as the condition of the bladder tissue, bladder contraction characteristics, donor age and the state of ureteric blood supply. Therefore, conclusions drawn from these reports may not be entirely accurate given these deficiencies. In order to better define the role of US post-transplantation, we reviewed our single center experience with the selective use of ureteral stenting only in high risk patients.

## METHODS

The clinical records of all patients transplanted at our center from July 2001 to July 2006 were reviewed with approval from the Institutional Review Board of SUNY, University at Buffalo, NY. Kidney recipients who lost their graft within 30 days post-transplant, patients with ureterojejunostomy or ureterocutanous anastomoses and those with intra-abdominal renal transplants were excluded from this study. A total of 301 patients were found eligible and were the subjects of this retrospective review. During the study period, it was the general practice to avoid placing US, unless patients were felt to be at high risk for urinary complications. Ureteral stents were selectively placed in 28 patients. Thirteen patients underwent ureteral stenting for a questionable blood supply to the ureter and 15 patients for an unhealthy appearing bladder or poor bladder capacity (four large neurogenic bladder with high residual (>100 mL), four thin wall bladder with severe adhesion of mucosa to the detrusor muscle and seven scarred small bladder (capacity <100 mL)).

All ureteroneocystostomies were completed using a Lich-Gregoir (external ureteroneocystostomy) or a single U-stitch technique. Briefly, the Lich-Gergoir technique is, after dissecting the bladder mucosa from the bladder muscle layer (detrusor muscle) and spatulating the ureter; the ureter is then circumferentially sewn by 6-0 PDS continuous sutures. Following this, the bladder muscular layer is re-approximated over the anastomoses and the ureter to produce an anti-reflux mechanism. The single U-stitch technique is performed by taking the hood of the spatulated ureter and securing it to the inside wall of the bladder with a single 4-0 PDS U-stitch. Then the myotomy is closed over the ureter to create anti-reflux tunnel. Single U-stitch technique was used only in patients with a healthy appearing bladder with a good capacity. In all patients with unhealthy appearing bladder wall, small bladder capacity (<100 mL) or scarred bladder, the Lich-Gregoir technique was used.

We retrospectively analyzed clinical outcomes in all patients with respect to urological complications such as leak, obstruction and stent-related problems including hematuria, infection, migration, irritative symptoms, stone formation, secondary obstruction from crusting, and complications from the stent removal procedure. The demographic characteristics of patients without US who developed urinary complications were compared to those who had an uncomplicated post-transplant course. 

Demographic and non-parametric outcome variables between groups were assessed using χ^2^ and Fisher’s exact tests. Unpaired *Student’s t* test was used for comparison of parametric data between the two groups. Kaplan-Meier estimation was used to study time to graft loss. A p<0.05 was considered statistically significant.

## RESULTS

Patient characteristics:

Of the 301 patients transplanted, 40 received a living related donor kidney, 16 had a living unrelated transplant and 245 were transplanted with a deceased donor kidney. The mean±SD age of donors was 40±16 years (range: 3 months to 79 years); the mean±SD age of recipients was 49±15 (range: 8–80) years. The mean±SD cold ischemia time was 15±9 hours; the mean±SD HLA mismatch was 3.6±1.7. Seventy-nine percent of patients received thymoglobulin (3-5 mg/kg total) induction therapy with the rest receiving an anti-IL2 receptor antibody. In 87% of patients, maintenance immunosuppression consisted of tacrolimus, mycophenolate mofetil and low-dose prednisone (5 mg/d by 30 days) the remaining patients received no steroids after 7 days. Only six (1.9%) patients received peri-operative prophylaxis antibiotics therapy due to a positive culture from the kidney bath. Ninety-two percent of patients received sulfamethoxazole/trimethoprim one single-strength tablet daily started at post-operative day two and continued for three months for *Pneumocystis carinii* pneumonia prophylaxis. The remaining patients received Dapsone or pentamidine aerosol for PCP prophylaxis due to sulfa allergy.

The overall 1- and 3-year patient and graft survival rates were 97% and 94%, and 97% and 83%, respectively. There were no surgically related post-transplant deaths. The majority of ureteral implantations were performed using the Lich-Gregoire technique (86%) with the rest being performed with the single U-stitch technique. The selection of ureteral implantation technique was based on surgeon preference. The Lich-Gregoire technique was used in all stented patients. It was practice to remove all US after 4–6 weeks post-transplantation. 

Both groups were similar in demographic characteristics with equal representation of patients with diabetes, hypertension, delayed graft function, acute rejection, HLA mismatches and cold ischemic time (Table 1). The two groups were also similar in recipient age and sex. 

**Table 1:** Patient demographics

Parameter	Stented	Non-stented
Number	28	249
Female %	52	48
Non-white%	31	34
Mean±SD age of donors (yr)	37±16	42±17
Mean±Sd age of recipients (yr)	49±15	49±14
Diabetes %	30	32
Mean±SD ischemia time (hrs)	17±7	18±7
HLA mismatch ±SD	3.4±1.6	3.1±1.3
Living donor %	14	17
Mean±SD hospital stay (d)	8.5±4.8	6.5 ±4.1

There were no significant differences between the two groups.

Stent complications: 

Of the 28 patients with US, six (21%) developed complications related to the stent. Two patients developed ureteral obstructions despite the presence of a US on post-operative days 3 and 29. Both obstructions resolved upon removal of the US. One patient developed an encrusted US because it was forgotten in place for 168 days. Removal of this US required surgical intervention and general anesthesia. Three patients developed early urinary tract infections within the first month, necessitating early stent removal. In contrast, none of the non-stented patients developed early (within first month post-transplant) urinary infections (p<0.001). There were no urinary leaks noted in the stented patients. Four (14%) patients experienced mild to moderate bladder discomfort and irritation that resolved after stent removal (Table 2). 

**Table 2:** Post-operative course in stented and non-stented groups

**Parameter**	**Overall**	**Stented** **(n=28)**	**Non- Stented** **(n=273)**	**p value**
Patient survival 1 year	97	98	96	NS
Patient survival 3 year	94	93	97	NS
Graft survival 1 year	97	97	97	NS
Graft survival 3 year*	87	86	87	NS
Urinary leaks: Overall	14 (4.6%)	0 (0%)	14 (5.1%)	NS
Single	8/253 (3.1%)	0 (0%)	8 (3.1%)	NS
Dual**	4/34 (15%)	0 (0%)	4 (12%)	NS
En-bloc**	2/14(14%)	0 (0%)	2 (14%)	NS
Early UTI <3 months (%)	3	3 (17.6)	0	<0.001
Early ureteral obstruction <3 m	6	2 (7%)	4 (1.4%)	0.08
Late ureteral obstruction > 3 m	14	2 (7%)	12 (4%)	0.07
Rejection rate %	27	33	27	NS
Mean±SD hospital stay (d)	7.9±4.6	8.5±4.1	6.5±4.1	NS
BK virus nephropathy	3	0	3	NS
CMV infection	18	0	18	NS

* Death censored.

** Dual and en-bloc kidneys were considered as two kidney transplants.

**Figure 1:**
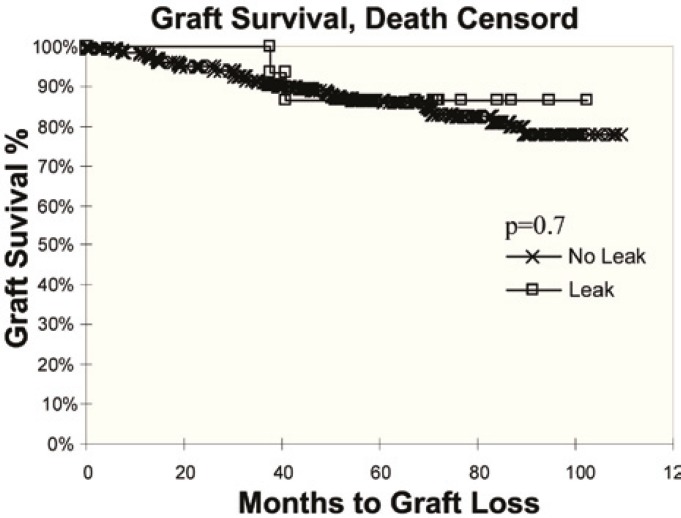
Graft survival, death censored, of patients with leak compared to those without leak

**Figure 2:**
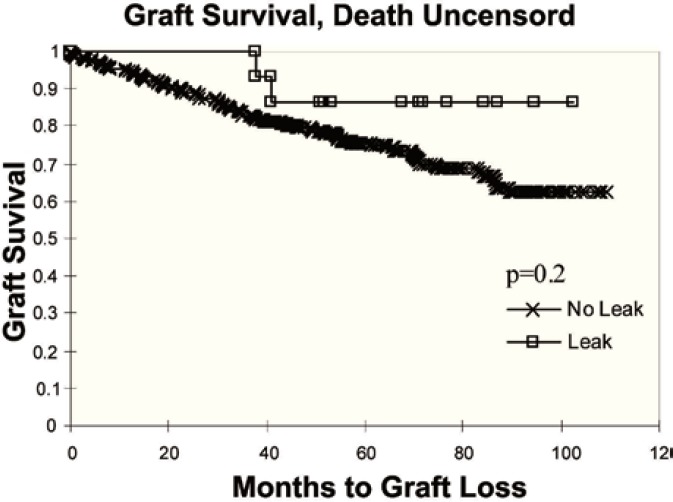
Graft survival, death uncensored comparing patients with leak vs no leak

Outcomes in non-stented patients:

Of the 273 non-stented patients, 12 (4.3%) developed a clinically significant urinary leak, which was not significantly different from the rate noted in patients with stent (0%; p=0.24). Urinary leaks occurred on average six days after transplantation. The risk for urinary leak is greater in dual kidney transplants irrespective of donor age compared to single kidney allografts (p=0.01). In addition, urinary leaks were more common when using older donor kidneys or expanded criteria donors and with the use of the single U-stitch technique (Table3). All patients with urinary leak underwent open exploration of the transplant and reimplantation of the ureter by Lich Gregoir technique with stenting. Exploration of these kidneys revealed necrosis of the ureteral tip in 10 patients and pinpoint anastomotic leak in two others. No graft was lost due to urinary complications. Short- and long-term graft survival was not significantly affected by urinary leak (Figures 1 and 2).

**Table 3:** Demographic of patients who experienced urinary leak vs. patient with no urinary leak

**Parameter**	**With urinary leak**	**Without urinary leak**	**p value**
Recipient number	14	259	
Mean±SD age of recipients	51±14	49±15	NS
Mean±SD age of donors	44±21	40±17	NS
Female %	46	37	NS
African American %	27	30	NS
Mean±SD weight	172±46	164±76	NS
DM %	27	30	NS
Mean±SD years of DM	20±10	20±5	NS
HLA match ±SD	3.4±1.6	2.2±2	NS
Mean±SD CIT	14±8	15±9	NS
DGF %	28	32	NS
Living donor (%)	7	17	NS
Dual kidney transplant %	33	5	0.0003
Extended criteria donor %	57	27	0.01
Median hospital stay	8±1.9	7±8	NS
Thymoglobulin induction %	66	76	NS

Urinary complication and CMV infection: 

Of the 301 patients in this study, 18 (5.9%) developed CMV infection, Of whom, four experienced tissue invasive CMV infection, three GI tract infections and one CMV retinitis. None of the 18 patients with CMV infection were stented. Only one patient with CMV infection experienced late ureteral stricture (p=0.45). 

Urinary complication and BKV infection: 

Of the 301 patients, only four (1.2%) developed BK virus infection, none of whom were stented; none of these four patients experienced early or late urinary complication. 

## DISCUSSION

As improvements in surgical techniques and immunosuppression therapies have been devised, the rate of surgical and urological complications following renal transplant have significantly fallen [1-6]. Our study, as well as many others [19-23], demonstrates that a urinary leak is the most common early urinary complication after renal transplantation. A urinary leak almost always occurs at the anastomotic site due to sloughing of the ureter tip in response to a compromised vascular supply. Urinary leaks may also occur due to technical errors; however, this is a rare cause of leaks [20, 21].

Karam, *et al*, [24] in their large series of 1629 patients reported a 3.2% rate of ureteral necrosis. Pathological examination of necrotic ureters revealed viral inclusion bodies in six (24%) patients, four of which were due to CMV virus and two showed BK viral inclusions. Other common causes of ureteral necrosis were arterial (n=4) and venous (n=16) thrombosis. Acute rejection was not observed in any of the specimens. Risk factors for ureteric necrosis included donor age, DGF and CMV infection. In our study, only seven ureteral tissue specimens were sent for histopathological evaluation after urinary leaks. All specimens showed tissue necrosis and cellular death. We did not appreciate any viral infection or rejection in our specimens. 

Many other factors such as ureteral anastomoses technique, bladder tissue condition (small and scarred due to long-term anuria), bladder contraction ability (neurogenic bladder in diabetic patients) and the blood supply to the ureter (damage to the ureteral blood supply during the kidney procurement) can also play a significant role in post-transplant ureteral complication. 

The transvesicular (Leadbetter-Politano) technique was reported to have less or comparable urinary leak rate compared to extravesical approach such as Lich-Gregoire or single stitch techniques due to longer submucosal tunneling, but it was associated with a higher rate of ureteral stenosis [25, 26]. In the current era of transplantation, majority of transplant surgeons prefer an extravesical techniques over the transvesical approach because it is faster, does not require a separate cystotomy, and needs less ureter length, therefore ensuring a better blood supply to the distal ureter. 

Some reports [27-29] showed less or comparable urinary complications with single U-stitch than other ureterocystostomy techniques. In contrary, others (4, 30, 31) found higher urinary complications with the single U-stitch technique compared to Lich-Gregoire technique and they concluded that single U-stitch technique should not be used in kidney transplantation. In our patients, 4 (9%) of 43 transplants with single U-stitch technique experienced a urinary leak compared to 8 (2.7%) patients in the Lich-Gregoire group (p=0.05). Despite the possibility of higher urinary leak when using the single U-stitch technique, this technique may be preferably used when transplanting kidney with very small ureter diameter such as in small pediatric donors to avoid ureteral stricture. 

Ureteral stenting can be used to treat small urinary leaks and can significantly reduce the rate of urinary leaks when used prophylactically. However, large necrosis of the distal ureter can present with significant urinary leak despite US. In our series, the incidence of urinary leaks without stenting was low (4.3%) and was not statistically increased compared to stented patients (p=0.24). We observed that urinary leaks were more frequent in kidneys from very Extended Criteria Donors (ECD) and dual kidney transplant. We suspect that the disrupted uretric blood supply due to severe arteriosclerosis in these old kidneys can be the major cause of higher urinary leaks in these patients. Therefore, we suggest that in case of dual transplants and when using kidneys from ECD donors routine stenting is appropriate. However, our study shows that the majority (97%) of the recipients of standard kidneys with normal bladders can enjoy kidney transplant without a urinary stent and without urinary leak. 

Ureteral stricture/obstruction is the second most common urinary complication after renal transplant [20, 21]. Early ureteral strictures or obstructions mostly occur due to technical error, twisting, kinking, external compression or severe mucosal edema. Some investigators reported that US reduce early post-operative ureteral stricture/obstruction [19, 23]. Others reported that the stent itself could be the cause of obstruction [33, 34]. In our group, 3 (11%) of 28 renal transplant recipients developed obstruction despite US due to stent malfunction and required early intervention to remove the stents. Of 273 renal transplants without stents 4 (1.4%) developed early ureteral obstruction: three due to severe mucosal edema and one on post-operative day 57 due to severe cellular rejection. All obstructions were treated successfully with percutaneous nephrostomy and antegrade insertion of ureteral stent.

Late ureteral stricture (after 3 months) is the most common late urinary complication of renal transplant. This complication is mostly caused by ureteral ischemia. However, other factors such as viral infection or acute and chronic allograft rejection can play a role in the late post-transplant ureteral strictures. We observed a trend towards increased late ureteral strictures in our stented group (17% *vs* 7%; p=0.08). This higher risk of late ureteral stricture in our stented group may be due to a selection bias since most patients receiving US were at high risk for this complication.

Karam, *et al*, [24] also reported that the number of CMV infections were higher in the group with ureteral necrosis (1.44 *vs* 1.23; p=0.001). In our study, despite the presence of CMV viremia and tissue invasive disease, there was no association between CMV and ureteral stenosis. 

Recently, Thomas, *et al* [35] from Johns Hopkins University reported that ureteral stenting might increase the risk of BK virus infection in the kidney transplant. In both univariate and multivariate logistic regression analysis adjusting for age, gender, deceased donor transplant, delayed graft function, tacrolimus and exposure to antibodies, the placement of ureteral stent at the time of the kidney transplantation was found to have statistically significant association with developing BK virus nephropathy. In our study, only 4 (1.2%) of 301 kidney transplant recipients developed BK virus nephropathy. None of these four patients were stented. This very low incidence of BK virus infection observed in our group when compared to other reports [36-39] may be due to our immunosuppressive therapy with its reduce steroid doses. However, we speculate that our low usage of US may also contribute to the low incidence of BK infection in our patients.

The other nuisance complications of ureteral stenting are bladder irritation and pelvic pain [13-17]. Although, these complications seem to be less frequent in the case of renal transplant compared to that in native ureters possibly due to the denervated state of the kidney transplant and early removal of the US. Four (14%) of our patients experienced such complications, which resolved after US removal. The bladder discomfort and pain in our patients might be due to the small, scarred and abnormal bladders of these patients. 

A potential concern with the use of selective stent placement is that without a careful record of who has been stented, patients may be missed and present with complications resulting from a forgotten stent. This unfortunate occurrence was seen in one of our patients, but this is well recognized in the literature [40-43].

The cost-effectiveness of routine compared to selective stenting was not addressed in this study. The major additional cost of not using stents routinely was the 12 surgeries required to correct urinary leaks in non-stented patients. In this study, 22 stents were needed to be placed to prevent one urinary leak. By broadening our selection criteria for US placement and by a more liberal use of the Lich-Gregoire technique, perhaps the rate of urinary leaks could be further lowered. In contrast, the benefits of not routinely stenting patients are difficult to calculate. The financial impact of increased hospital admissions due to urinary infections and placement of nephrostomies for late strictures needs to be considered. Furthermore, the potential benefits of reduced BK infections and graft loss in non-stented patients is only now being identified. 

In summary, the medical literature regarding the routine use of US remains inconclusive. Since clinical trials in this field rarely describe in detail the population studied and therapies used in terms of patient demographics, donor and recipient characteristics, surgical techniques, immunosuppressive strategies and recipient bladder function, it is difficult to draw conclusions about the practice of using US for all patients. Only a well-designed prospective multicenter trial comparing selective *vs* universal stenting can assess the benefits and costs of this procedure. Any study will need to include a quality of life assessment, which would help clarify the true costs to the patient of routine stenting. 

An inherent deficiency of our study is the small number of patients in the stented group compared to the non-stented group. However, our study demonstrates that the vast majority (97%) of kidney transplant patients, particularly those who receive standard kidneys without evidence of damaged ureteral blood supply and who have no evidence of bladder dysfunction can be successfully transplanted without the routine use of US.
